# ATM’s Role in the Repair of DNA Double-Strand Breaks

**DOI:** 10.3390/genes12091370

**Published:** 2021-08-31

**Authors:** Atsushi Shibata, Penny A. Jeggo

**Affiliations:** 1 Signal Transduction Program, Gunma University Initiative for Advanced Research (GIAR), Gunma University, Gunma 371-8511, Japan; 2 Genome Damage and Stability Centre, School of Life Sciences, University of Sussex, Brighton BN1 9RQ, UK; p.a.jeggo@sussex.ac.uk

**Keywords:** DNA double-strand break, ionizing radiation, ATM, non-homologous end joining, homologous recombination

## Abstract

Ataxia telangiectasia mutated (ATM) is a central kinase that activates an extensive network of responses to cellular stress via a signaling role. ATM is activated by DNA double strand breaks (DSBs) and by oxidative stress, subsequently phosphorylating a plethora of target proteins. In the last several decades, newly developed molecular biological techniques have uncovered multiple roles of ATM in response to DNA damage—e.g., DSB repair, cell cycle checkpoint arrest, apoptosis, and transcription arrest. Combinational dysfunction of these stress responses impairs the accuracy of repair, consequently leading to dramatic sensitivity to ionizing radiation (IR) in ataxia telangiectasia (A-T) cells. In this review, we summarize the roles of ATM that focus on DSB repair.

## 1. Introduction

Ataxia telangiectasia (A-T) was identified as a human disorder displaying radiosensitivity at both the cellular and clinical level in 1975 [1], and was amongst the first of the DNA damage response disorders to be characterised. A-T has a broad clinical manifestation with individuals displaying progressive cerebellar degeneration, immunodeficiency and cancer predisposition. However, although dramatic sensitivity to ionising radiation (IR) and to radiomimetic drugs was evident in that first and additional early reports with cells derived from A-T individuals displaying marked sensitivity to cell killing and to chromosome aberrations, subsequent studies failed to reveal any significant defect in the repair of DNA double strand breaks (DSBs), the main lethal lesion induced by IR exposure [2]. The characterisation of a phenotype termed ‘radioresistant DNA synthesis’, which had been amongst the earliest identified defects in A-T cells [3], raised the possibility that the inability to respond to DNA damage rather than a defect in the ability to repair the damage was at the root of the radiosensitivity. Fuel was added to the conundrum when A-T was characterised as having a p53-dependent G1/S cell cycle checkpoint defect, resulting in its categorisation as the first human cell cycle checkpoint disorder [4], with both a G1/S and an intra-S phase checkpoint defect. A broad array of further phenotypes were observed in the ensuing years included defective meiotic recombination. Finally, in 1995 the causal genetic defect, the ataxia telangiectasia mutated (ATM) gene, was identified as a phosphatidylinositol 3-kinase (PI3K) family member, which were known to play significant roles in signal transduction [5]. Subsequently, ATM was shown to be a protein kinase rather than a lipid kinase and classified as a PI3K-like kinase (PIKK). These important findings helped to explain the broad phenotypes of A-T cells and the diverse clinical manifestation of the disorder. It is now appreciated that ATM, which is activated both by DNA DSBs and by oxidative stress, has a vast array of substrates and, in response to DNA damage or oxidative stress, initiates a plethora of responses. The co-ordination of these responses optimises the repair of DSBs in the context of chromatin structure and the interface with other DNA metabolic processes, such as transcription. Failure to initiate this broad range of responses impacts upon DSB repair in complex ways, which we detail here.

As techniques to monitor DNA DSB repair improved and primary rather than immortalised cells were used, A-T was identified as having a subtle but significant defect in the repair of a subset of radiation induced DSBs in non-cycling cells [6]. However, it is also evident that additional consequences of the signalling defect in A-T cells can confer deficiences in the repair of DSBs, with the defect often being manifest as a decrease in the fidelity of repair rather than affecting the overall level of repair. In some cases, mis-repaired DSBs may be tolerated and survival can ensue. However, frequently, mis-repaired DSBs, such as chromosome rearrangements or large deletions, will be lethal. Thus, although the outcome of misrepair versus no repair may be similar, simply assessing DSB repair levels may not provide a good assessment of DSB repair. In this review, we will focus on the roles that ATM plays to ensure efficient repair (of both the level and fidelity) of DSBs. We will focus on the role of ATM in regulating four major processes, cell cycle checkpoint arrest, the arrest of transcription in the vicinity of DSBs, the repair of a specific subset of DSBs, and its influence on DSB repair pathway choice, largely involving its regulation of DSB end-resection. However, at the core of most of these processes, is the role that ATM plays in regulating chromatin structure at the DSB site. We will first discuss how ATM is activated and how it influences chromatin structure. We will then consider how the four responses, when perturbed, impede the level of DSB repair and its fidelity. Finally, we will evaluate how these defects might contribute to the clinical manifestation of A-T.

## 2. ATM Activation

**Figure 1:**
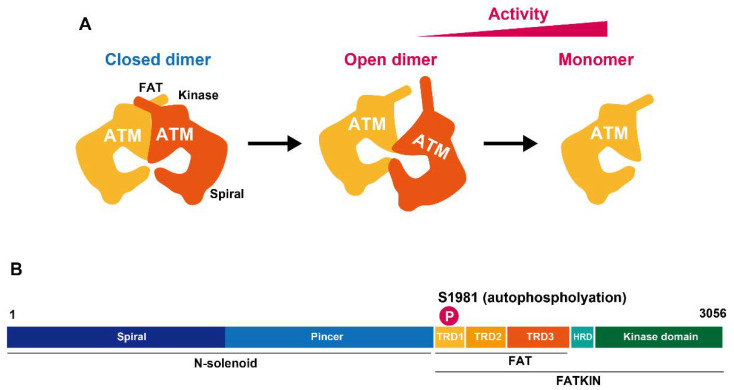
ATM activation after DSB formation. (**A**) ATM structure is associated with the kinase activity. The monomeric form has greater kinase activity than the dimeric form. (**B**) A two-dimensional ATM structure with some of ATM’s relevant domains indicated.

The primary activation mode of ATM kinase activity is its dissociation from a dimeric form by autophosphorylation at Ser1981. In 2003, Kastan’s group elegantly demonstrated that the ATM dimer is autophosphorylated in response to DNA damage, subsequently the active form of an ATM monomer promotes the downstream phosphorylation and interaction with ATM partners [7]. More recently, crystal structure analysis uncovered the high-resolution structure of ATM dimers and monomers. Single-particle electron microscopy demonstrated that ATM active sites are buried, restricting access of the substrates to these sites in the dimeric structure [8]. Furthermore, studies using electron cryomicroscopy (cryo-EM) suggest that the ATM dimeric structure has two distinct dynamic forms, i.e., closed and open dimers (Figure 1) [9,10,11]. In the closed state, the PIKK regulatory domain blocks the peptide substrate–binding site. In contrast, the active site is held in this closed conformation by interaction with a long helical hairpin in the TRD3 (tetratricopeptide repeats domain 3) domain of the symmetry-related molecule; this suggests that the open conformation may be more active. Nevertheless, the kinase activity of the monomer is ~10-fold higher than the open dimeric form [11]. Although the precise in vivo regulation is unknown, it has been proposed that the open dimer is a transition state between the closed dimer and monomer states. Alternatively, it could be a structure to fine-tune ATM activity dependent on the biological situation. 

**Figure 2:**
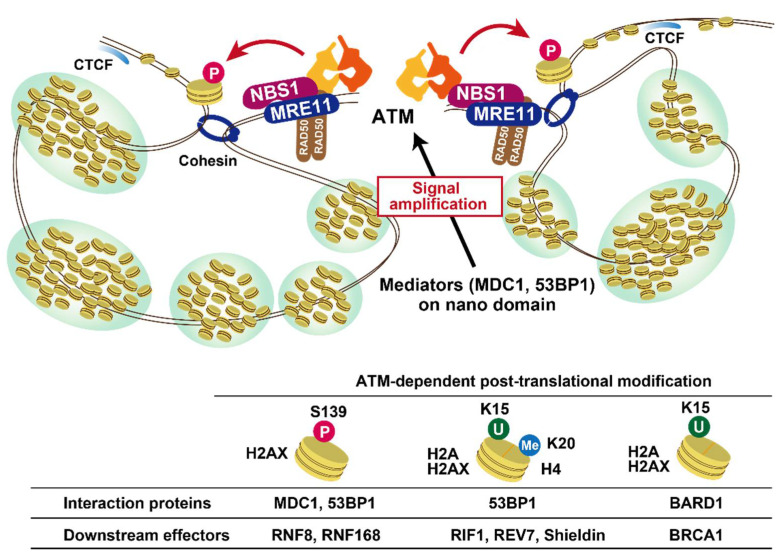
ATM activation at DSB sites. ATM phosphorylates H2AX at S139, this, in turn, promotes the recruitment of MDC1, which, in its own turn, facilitates the RNF8/RNF168-dependent ubiquitination of H2AX at K15. These signals recruit 53BP1 to form nano domains, which are visualized as nano foci. ATM-dependent post-translational modifications are shown in the bottom panel. The interacting proteins and downstream effectors are described in more detail in the text. CTCF: CCCTC-Binding Factor.

Biologically, several different activation modes have been reported. A DSB is the primary form of triggering ATM activation by dimer dissociation. The MRE11/RAD50/NBS1 (MRN) complex acts as the DSB sensor, thereby recruiting ATM at DSB sites, particularly by an interaction between ATM and Nijmegen breakage syndrome protein 1 (NBS1), resulting in further accumulation of ATM signaling and positive feedback [12,13,14] (Figure 2). ATM becomes hyperactive in DNA-dependent protein kinase catalytic subunit (DNA-PKcs) deficient cells or cells treated with a DNA-PK inhibitor, suggesting that DNA-PKcs counteracts the over-activation of ATM kinase activity under physiological conditions [15]. This mechanism is considered to maintain normal cell growth by preventing unnecessary apoptotic pathway activation. A recent study using high-throughput chromosome conformation capture (Hi-C) and chromatin immunoprecipitation sequencing (ChIP-Seq) analyses showed that the signal of ATM pS1981 (a marker of autophosphorylation and ATM activation) shows a sharp peak at the DSB site compared with the γH2AX (a phosphorylation form of H2AX) signal, a downstream substrate of ATM, suggesting that the active form of ATM is predominantly located near DSBs [16]. The proposed model suggests that the locally recruited ATM to DSB ends phosphorylates H2AX. Concomitantly, ATM activates a cohesion-dependent loop extrusion that further promotes H2AX phosphorylation along the chromatin until the loop extrusion is blocked at the topologically associating domain boundary element [16,17] (Figure 2). Thus, ATM can reach and phosphorylate multiple targets along the chromatin even if localization of ATM near a DSB end is required to sustain its activation (see also the discussion of ATM localization in Section 3).

In addition, ATM can be activated under conditions of oxidative stress following the formation of dimers via disulfide-crosslinks [18]. This mode of ATM activation is genetically separable from the process described above, requires distinct sites within ATM and is MRN-independent. Whereas loss of DNA damage-induced ATM activation confers loss of cell viability, failure to activate checkpoint arrest and end-resection, loss of oxidative activation has minimal impact on these outcomes but impedes ATM-mediated checkpoint response after oxidative response, deficiency in mitochondrial function and autophagy [19]. ATM has also been reported to be activated in response to the alteration of chromatin structure without DNA damage although whether this requires MRN and the DNA damage or oxidative damage sites has not been established [7]. In response to DSBs, ATM is exported from the nucleus and can stimulate NF-κB–dependent signal transduction [20]. Hence, ATM has multiple modes for the activation of its kinase activity in response to DSBs and cellular stress. These diverse activation modes may be critical to select the best responses by targeting selective substrates from the multitudinous ATM substrates in response to distinct types of genotoxic stress. Since here, we will discuss the impact on DSB repair, we will focus on the canonical activation of ATM at DSBs.

## 3. Role of ATM in DNA Damage Signalling

ATM lies at the heart of a signal transduction process alerting cells to the presence of DSBs. The most significant aspect of this is to alter the chromatin in the DSB vicinity. For this process, multiple repair mediators—mediator of DNA damage checkpoint protein 1 (MDC1), RING finger ubiquitin E3 ligase (RNF8), RING finger 168 (RNF168), breast cancer susceptibility protein 1 (BRCA1), and p53 binding protein 1 (53BP1)—are recruited in an ATM-dependent manner (see the details in our previous reviews [21,22,23]). As damage response mediators, MDC1 and 53BP1 contribute to the amplification of ATM activity surrounding DSBs. MDC1 binds to γH2AX and the recruitment is identified as foci [24], suggesting that the distribution of MDC1 is also estimated to be a million base pairs around DSBs. In addition, MDC1 also interacts with MRE11, which also promotes the tethering of MRN, and also ATM [25,26]. Furthermore, MDC1 interacts with RNF8 via ATM-dependent MDC1 phosphorylation [27,28,29]. This signal cascade promotes the formation of K63-linked ubiquitin chains in an RNF8/RNF168-dependent manner and subsequently the recruitment of 53BP1 on chromatin. In addition to MDC1, 53BP1 interacts with the MRN complex, which promotes the activation of ATM at DSB sites [30]. Given that the DNA damage response mediators, MDC1, RNF8/RNF168, and 53BP1, play major roles in ATM-dependent signal expansion and that deficient cells have smaller ATM foci [31], it is unclear how this previous model of ATM recruitment and foci expansion is reconciled with the recent findings discussed above, that activated ATM is only localised at the DSB site. One possibility is that the initial activation of ATM may occur at close proximity to DSB ends, and subsequently ATM can be recruited in a mediator-dependent manner along the chromatin, with the signal being amplified onto the scaffold proteins. Such a model is possible if the second phase of ATM binding is not detected by ChIP if it is not tightly bound to chromatin. An alternative possibility is that the mediators promote ATM turnover or tethering at the DSB site but not when distant from the DSB site.

## 4. Cell Cycle Checkpoint Arrest

An important role of ATM is the activation of cell cycle checkpoint arrest (G1/S, intra-S and G2/M checkpoint arrest). In some cell types, apoptosis can also be activated if an excessive amount of DNA damage is induced. Central to checkpoint activation is the ATM-dependent phosphorylation of Chk2 (see Bartek & Lukas for a review) [32]. Although phosphorylated Chk2 spreads cell-wide [33], the initial phosphorylation event arises at γH2AX foci and thus requires all the ATM-dependent signal transduction proteins described above for optimal activation [34]. The processes of checkpoint arrest have been well described previously and will not be detailed here [32]. The impact of checkpoint arrest on DSB repair has also been discussed [35]. Importantly, the activation of checkpoint arrest provides time to allow DSB repair to be completed prior to the onset of replication or mitosis, and failure to arrest efficiently can dramatically impede the fidelity of DSB repair. It is important to note, however, that this process is only significant for cycling cells and will not impact upon G0 arrested cells, which actually represents the majority of cells in our body. This demonstrates that, whilst cell cycle checkpoint arrest is an important process enhancing survival post irradiation, it is certainly not the only ATM-regulated process influencing radiosensitivity.

## 5. Impact of ATM Signaling on Transcription

In parallel to cell cycle arrest and DSB repair, the transcription machinery is also arrested to prevent aberrant mRNA synthesis at damaged transcription regulatory sequences. ATM has a critical role in preventing transcription after DSB formation by RNA polymerase II (RNAPII) that has responsibility for mRNA synthesis at gene loci, and RNA polymerase I that synthesizes ribosomal RNA [36,37]. ATM-dependent transcriptional silencing occurs via RNF8 and RNF168 dependent K63-linked ubiquitination. Histone H2A ubiquitination which requires ATM activity also mediates RNAPII transcription silencing. In addition, ATM phosphorylates BRG1-associated factor 180 (BAF180), a subunit of polybromo-associated BAF complex (PBAF) that is a chromatin remodeler, to suppress RNAPII elongation [38]. Because cohesin, which regulates chromatin looping, is also required in this axis [39], the dynamic change of chromatin structure may affect the overall transcription machinery. However, the silencing occurs in cis to DNA damage, i.e., possibly the local relationship between damage and transcription repression is localised and possibly on the same chromosome. In G1 phase, failure to activate transcriptional arrest has been shown to delay the rate of DSB repair without affecting the final level of repair [38]. However, loss of transcriptional arrest enhances chromosomal rearrangements suggesting an impact on the fidelity of repair [39]. This process, however, affects a minor, although very important, subset of DSBs.

## 6. Role of ATM in the Repair of a Subset of DSBs: Role of Chromatin Remodeling

**Figure 3:**
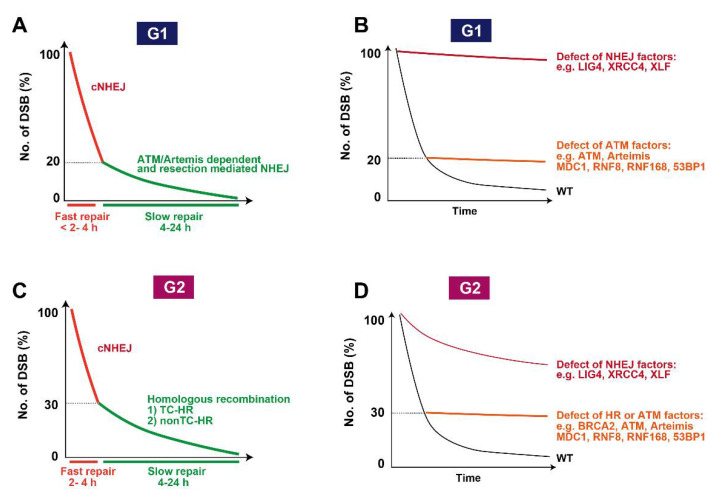
DSB repair kinetics after <1–5 Gy. (**A**) DSB repair kinetics in G1 phase. (**B**) An example of repair kinetics in defective NHEJ or ATM-related factors is shown. (**C**) DSB repair kinetics in G2 phase. (**D**) An example of repair kinetics in defective NHEJ, HR, or ATM-related factors is shown.

Although initial studies suggested that the level of DSB repair was normal in A-T deficient cells, accumulating evidence clearly shows that lack of ATM kinase activity causes a defined DSB repair defect [6]. Nonetheless, the magnitude of the DSB repair defect is not as big as expected considering that ATM-deficient cells exhibit strong radio-sensitivity equivalent to cells defective in non-homologous end joining (NHEJ) [40]. In 2004, the Jeggo and Lobrich groups applied γH2AX foci analysis to measure DSB repair capability by enumerating γH2AX foci [6]. Since γH2AX foci analysis is a more sensitive assay compared with the pre-existing physical techniques such as the neural comet assay or pulsed-field gel electrophoresis, the analysis uncovered the existence of an ATM-dependent DSB repair fraction, which is approximately 15–20% of the total induced DSBs in irradiated G0/G1 cells (Figure 3A,B). In addition, depletion of the other downstream factors such as 53BP1, MDC1, and RNF8/RNF168 show a similar repair defect level, whereas a defect in core NHEJ factors such as X-ray cross complementing gene 4 (XRCC4), XRCC4-like-factor (XLF), or DNA ligase IV (LIG4) exhibit more substantial repair defects (Figure 3B) [6,31]. Interestingly, depletion of heterochromatin factors, KRAB-associated protein 1 (KAP-1), heterochromatin protein 1 (HP1), or histone deacetylase 1/2 (HDAC1/2), alleviates the repair defect in ATM-deficient cells [31,41]. ATM-dependent KAP-1 phosphorylation at Ser824 [42] promotes the dispersal of the nucleosome remodeler chromodomain helicase DNA-binding 3 (CHD3) from DSBs, which triggers chromatin relaxation at DSB sites [43] (see the details in our previous review [44]). In this axis, requirement for direct interaction between 53BP1 and γH2AX via the BRCA1 C-Terminal (BRCT) domain in 53BP1 was identified to amplify the ATM-pKAP1 signaling for ATM-dependent DSB repair [45]. In addition, suppressor of cancer cell invasion (SCAI), another 53BP1 interactor whose interaction is ATM kinase-dependent, facilitates this process [46]. Together, these studies suggest a role for ATM in coordinating chromatin remodeling during the repair process. Interestingly, cells defective in Artemis also show similar levels of defective DSB repair to ATM-deficient cells. Artemis-dependent DSB repair requires MRE11 exonuclease activity and CtBP-interacting protein (CtIP), suggesting that the subset of DSBs that undergo resection prior to the rejoining have a requirement for Artemis and ATM [47]. Importantly, ATM inhibition does not show an additive repair defect in Artemis cells, indicating that ATM and Artemis play a role in DSB repair in the same axis. Since it is unlikely that Artemis directly participates in the chromatin remodeling event, it is still unknown how these two factors facilitate the repair process of DSBs in association with chromatin remodeling. 

## 7. Roles of ATM in DSB End Resection Influencing Pathway Choice

ATM plays a role in DSB end resection, which affects the fidelity of DSB repair. The dysregulation of resection impairs the progression of homologous recombination (HR) in S/G2 phase. The rejoining by these inefficiently resected DSBs by NHEJ likely leads to deletion mutations. In S/G2 phase, the signaling machinery switches from ATM to ATR in concert with the progression of resection because ATM is primarily activated at unresected DSB ends. In contrast, ATR is activated on ssDNA-replication protein A (RPA) following resection [48,49]. Therefore, the temporal switching of the two kinases occurs following the progression of resection; however, ATM seems to be involved throughout the process of HR, i.e., from initiation of resection to termination of resection (or restriction of excessive resection). ATM is also required for the repair of 20–30% of DSBs after IR in S/G2, subtly greater than the requirement for ATM in G1 phase (Figure 3C). Depletion of breast cancer susceptibility gene 2 (BRCA2), which is an essential factor for HR by RAD51 recombinase (RAD51) recruitment, shows a defect in the repair of ~30% of DSBs in G2 phase. Similarly, ATM inhibition shows the same level of DSB repair defects in G2 phase [50] (Figure 3D). 

**Figure 4:**
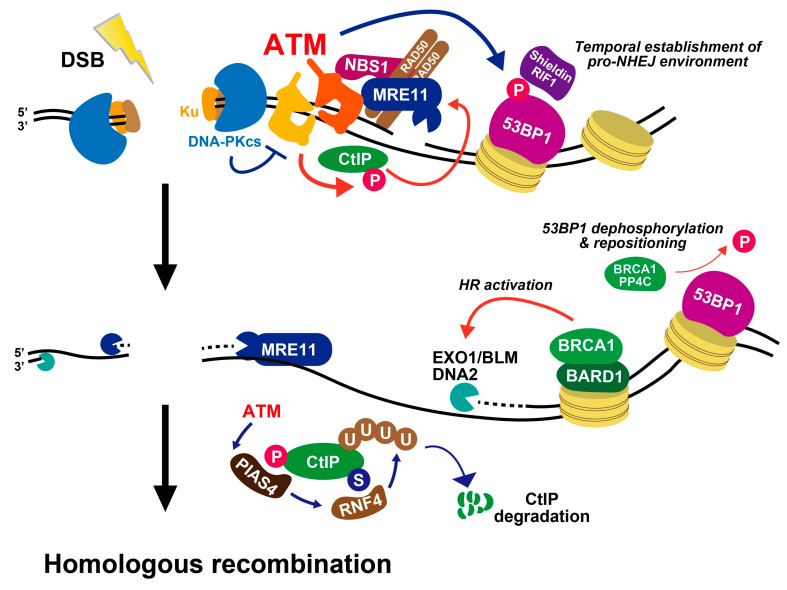
Roles of ATM during DSB end resection in G2 phase. See the text for details.

In response to DSB induction in S/G2 phase, ATM is rapidly activated, similar to the situation in G1 phase inducing the recruitment of DDR responders and promoting foci formation such as γH2AX, MDC1, and 53BP1, followed by downstream signaling. 53BP1 hyper-phosphorylation, strictly dependent on ATM, occurs in S/G2 and G1 phases [51]. The hyper-phosphorylation of 53BP1 promotes the recruitment of replication timing regulatory factor 1 (RIF1), REV7, and Shieldin complexes that limit resection progression to promote NHEJ by Polα primase recruitment, possibly to fill in the ssDNA gap [52,53]. Such a pro-NHEJ environment is turned into a pro-HR environment following 53BP1 dephosphorylation facilitated by BRCA1-protein phosphatase 4 catalytic subunit (PP4C) in S/G2 phase [51] (Figure 4). In parallel, but possibly sequentially, ATM phosphorylates CtIP, a central factor initiating resection, which activates MRE11 endonuclease activity [54], generating a nick (or nicks) to initiate bidirectional exonuclease dependent resection by MRE11 and Exonuclease 1 (EXO1)/BLM/DNA2 exonuclease activities [55,56,57] (Figure 4). Activated CtIP following its phosphorylation at Ser664/679/745 is required for resection and HR [58,59]. This nicking commonly occurs to initiate resection at transcription-dependent and independent DSBs [59,60]. However, it is unknown exactly how these phosphorylation sites in CtIP affect the endonucleolytic activities although data suggest that phosphorylation promotes the recruitment or maintenance of CtIP at DSB sites [59,60]. In addition to these sites, a total of eight SQ/TQ sites in CtIP are potentially ATM-dependent phosphorylation sites [58]. Particularly, disruption of the T859 site, with intact Ser664/679/745 residues impairs resection and HR. ATM-dependent CtIP phosphorylation occurs after DSB formation. ATM-dependent phosphorylation event requires CDK-dependent phosphorylation of CtIP, suggesting that CtIP is effectively phosphorylated by ATM in S/G2 phase to promote resection after DNA damage [58,61]. The phosphorylation promotes interaction between CtIP and NBS1, which helps (CtIP)-dependent endonucleolytic incision [62]. Following the progression of CtIP-dependent resection, 53BP1 dephosphorylation releases RIF1, and its downstream factors from chromatin changes to promote a pro-HR environment, i.e., HR-associated BRCA1 complex and exonucleases (EXO1/DNA2/BLM) are recruited following 53BP1 repositioning, which generates a 53BP1-free chromatin area in the immediate DSB vicinity [51,63,64] (Figure 4). The role of ATM-dependent chromatin remodeling in HR is also proposed because the defective DSB repair in an ATM or 53BP1 deficient background is rescued by KAP-1 depletion in irradiated G2 cells [59,65]. However, under ATM inhibition, because ATM is essential for the initiation of resection, NHEJ can repair the DSBs, i.e., DSBs are repaired by NHEJ in ATM and KAP-1 double depleted cells. However, again, the impact of pre-existing heterochromatin or active chromatin remodeling is still under debate.

In parallel to the progression of resection, ATM also controls CtIP activity to restrict excessive resection. A recent study showed that protein inhibitor of activated STAT4 (PIAS4) dependent SUMOylation of CtIP promotes RNF4 dependent CtIP ubiquitination, which leads to its degradation [66] (Figure 4). ATM-dependent CtIP phosphorylation precedes CtIP-SUMOylation and the lack of the phosphorylation impairs the downstream CtIP degradation. Impaired CtIP ubiquitination results in excessive resection and defective HR. Thus, CtIP degradation in the ATM-dependent signal cascade may serve to restrict excessive resection during HR. In addition and interestingly, the inhibition of ATM after the resection step slows down RAD51 removal from the chromatin, suggesting that ATM plays a role in promoting RAD51 displacement [67]. Furthermore, ATM also phosphorylates ubiquilin-4 (UBQLN4), a proteasome shuttling factor, which promotes the ubiquitylation of MRE11 to fine-tune the magnitude of resection [68]. Thus, even if ATM is activated at the beginning of the DSB repair process, the phosphorylation events comprehensively control the overall HR process.

The regulation of DNA-replication-fork-associated single-ended DSBs (seDSBs) in S phase is different from two-ended DSBs because seDSBs must be directed toward HR because the lack of a DSB counterpart at seDSBs leads to chromosomal translocation if NHEJ is used. Consistent with this notion, lack of ATM activity leads to toxic LIG4-mediated chromosome fusions after DNA-replication-fork-associated seDSBs [69]. Appropriate removal of NHEJ components (Ku/DNA-PKcs) is required to direct the repair pathway towards HR at seDSB. The removal of the NHEJ component is achieved by MRE11/CtIP nuclease activities [70]. At seDSB ends, ATM phosphorylates DNA-PKcs, and phosphorylation at the ABCDE cluster of DNA-PKcs promotes the release of Ku from DSB ends in an MRE11/CtIP-dependent manner [71]. Following the rapid removal of NHEJ components, it is likely that a similar mechanism is used between seDSBs and two-ended DSBs.

To summarise this section, ATM has a critical role in regulating resection at DSBs, which arises via complex regulation of multiple substrates and critically depends on the regulation of chromatin structure at the DSB site. Not surprisingly, this impact of ATM affects DSBs in diverse ways. One affect is a failure to activate HR in G2 phase. However, although less dramatic, resection also arises in G1 phase, impacting upon repair via NHEJ. Here, two forms of NHEJ have been described, resection independent and dependent, and the latter is ATM-dependent in G1 phase [47]. Failure to appropriately regulate resection will predominantly affect the fidelity of DSB repair.

## 8. Summary

**Figure 5:**
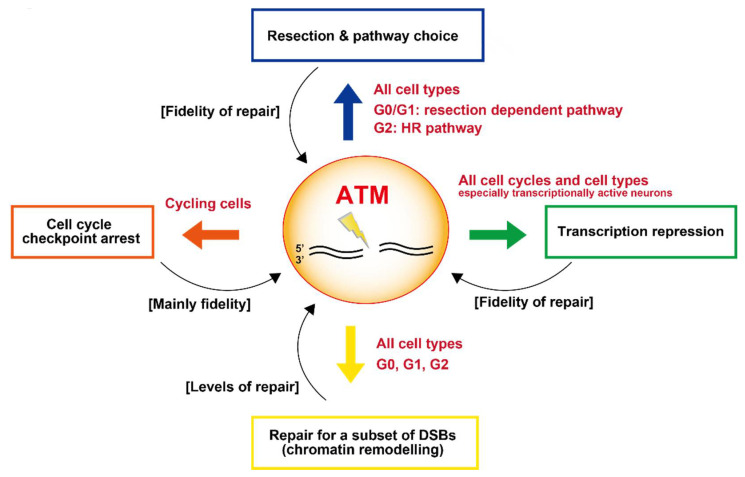
Summary highlighting the 4 pathways discussed in the text by which ATM influences the repair of DSBs. The impact on the level or fidelity of DSB repair in each pathway is shown by brackets; the type of cell affected is highlighted in red. Additional impacts of ATM, such as its role in regulating apoptosis, may also influence survival levels in response to DSBs but here we focus on pathways that influence DSB repair.

The details above reveal the significant functions that ATM has in determining the response to DSBs and its influence on how they are repaired. Here, we describe how ATM is essential for the repair of a small fraction of DSBs, its requirement for cell cycle checkpoint arrest, transcriptional repression and resection, and hence an influence on pathway choice, at DSBs (Figure 5). As briefly mentioned, ATM can also influence NF-κB signaling following its export to the cytoplasm. These impacts can influence both the level and fidelity of DSB repair. Indeed, one of the early hallmarks of ATM-deficient cells was a profound increase in chromosomal aberrations following IR exposure. A major question that arises is how these different consequences of ATM loss are manifest. For example, which of these roles of ATM has the biggest influence on radiosensitivity or impact clinically. Unfortunately, since all these processes interface and intertwine, it is difficult to unravel their varying contributions. However, it is important to appreciate that the precise tissue or cell type under analysis as well as cell cycle status is a major determinant. Stem cells differ from differentiated cells, for example; lymphocytes differ from fibroblasts. Cell cycle checkpoint arrest will not be influential in non-cycling cells. Artemis deficiency, which is epistatic with ATM loss, confers significant radiosensitivity in quiescent cells, demonstrating the significance of the modest DSB repair defect in A-T cells [6]. Some neuronal cells are highly transcriptionally active, potentially rendering transcription repression at DSBs of significance. Cytoplasmic signaling to NF-κB may be more significant in cells that readily undergo apoptosis, such as progenitor cells. Clinically, ATM deficiency causes ataxia telangiectasia, a broad-based multi-system disorder. Why ATM deficiency specifically results in marked loss of Purkinje cells and cerebellar function remains unclear. Why telangiectasia arises is not well explained. Since we now have a reasonable understanding of the various steps regulated by ATM during DSB repair, a current challenge is to understand which processes are important in which situation.

## Data Availability

Not applicable.
